# Incretins in patients with rheumatoid arthritis

**DOI:** 10.1186/s13075-017-1431-9

**Published:** 2017-10-17

**Authors:** Beatriz Tejera-Segura, Raquel López-Mejías, María Jesús Domínguez-Luis, Antonia M. de Vera-González, Alejandra González-Delgado, Begoña Ubilla, José M. Olmos, José L. Hernández, Miguel A. González-Gay, Iván Ferraz-Amaro

**Affiliations:** 1 Division of Rheumatology, Hospital Universitario de Canarias, 38320 Tenerife, Spain; 2 Epidemiology, Genetics and Atherosclerosis Research Group on Systemic Inflammatory Diseases, Hospital Universitario Marqués de Valdecilla, Instituto de Investigación Marqués de Valdecilla (IDIVAL), Santander, Spain; 3 Central Laboratory Division, Hospital Universitario de Canarias, Tenerife, Spain; 4 Division of Internal Medicine, Hospital Universitario Marqués de Valdecilla, Universidad de Cantabria, Santander, Spain; 5 Division of Rheumatology, Hospital Universitario Marqués de Valdecilla, Instituto de Investigación Marqués de Valdecilla (IDIVAL), Santander, Spain; 6 School of Medicine, University of Cantabria, Santander, Spain; 7 Cardiovascular Pathophysiology and Genomics Research Unit, School of Physiology, Faculty of Health Sciences, University of the Witwatersrand, Johannesburg, South Africa

**Keywords:** Insulin resistance, Incretins, Rheumatoid arthritis

## Abstract

**Background:**

The precise mechanism linking systemic inflammation with insulin resistance (IR) in rheumatoid arthritis (RA) remains elusive. In the present study, we determined whether the incretin-insulin axis and incretin effect are disrupted in patients with RA and if they are related to the IR found in these patients.

**Methods:**

We conducted a cross-sectional study that encompassed 361 subjects without diabetes, 151 patients with RA, and 210 sex-matched control subjects. Insulin, C-peptide, glucagon-like peptide-1 (GLP-1), gastric inhibitory polypeptide (GIP), dipeptidyl peptidase 4 (DPP-4) soluble form, and IR indexes by homeostatic model assessment (HOMA2) were assessed. A multivariable analysis adjusted for IR-related factors was performed. Additionally, ten patients and ten control subjects underwent a 566-kcal meal test so that we could further study the postprandial differences of these molecules between patients and control subjects.

**Results:**

Insulin, C-peptide, and HOMA2-IR indexes were higher in patients than in control subjects. This was also the case for GLP-1 (0.49 ± 1.28 vs. 0.71 ± 0.22 ng/ml, *p* = 0.000) and GIP (0.37 ± 0.40 vs. 1.78 ± 0.51 ng/ml, *p* = 0.000). These differences remained significant after multivariable adjustment including glucocorticoid intake. Disease Activity Score in 28 joints with erythrocyte sedimentation rate (β coefficient 46, 95% CI 6–87, *p* = 0.026) and Clinical Disease Activity Index (β coefficient 7.74, 95% CI 1.29–14.20, *p* = 0.019) were associated with DPP-4 serum levels. GLP-1 positively correlated with β-cell function (HOMA2 of β-cell production calculated with C-peptide) in patients but not in control subjects (interaction *p* = 0.003). The meal test in patients with RA revealed a higher total and late response AUC for glucose response, a later maximal response of C-peptide, and a flatter curve in GIP response.

**Conclusions:**

The incretin-insulin axis, both during fasting and postprandial, is impaired in patients with RA.

**Electronic supplementary material:**

The online version of this article (doi:10.1186/s13075-017-1431-9) contains supplementary material, which is available to authorized users.

## Background

The concept that oral nutrient (glucose) administration promotes a much greater degree of insulin secretion than a parenteral isoglycemic glucose infusion underlies the incretin effect, namely the existence of gut-derived factors that enhance glucose-stimulated insulin secretion from the islet β cell. This phenomenon is estimated to account for approximately 50–70% of the total insulin secreted following oral glucose administration. To date, gastric inhibitory polypeptide (GIP) [1] and glucagon-like peptide-1 (GLP-1) [2] fulfill the definition of an incretin hormone in humans.

Furthermore, several studies have shown that these two peptides potentiate glucose-stimulated insulin secretion in an additive manner, likely contributing equally to the incretin effect and together fully accounting for most of the incretin effect in humans. GIP and GLP-1 are degraded by dipeptidyl peptidase 4 (DPP-4) [3], which is a membrane-associated peptidase widely distributed throughout numerous tissues. DPP-4 also exists as a soluble circulating form in plasma, and significant DPP-4-like activity is detectable in plasma from humans. Several studies have confirmed that DPP-4-mediated inactivation of these peptides is a critical control mechanism for regulating the biological activity of both GIP and GLP-1 in vivo in humans [4]. This arc of discovery has led to newly approved antidiabetic therapies during the last decade: GLP1 analogues (exenatide, liraglutide) and DPP-4 inhibitors (saxagliptin, sitagliptin, vildagliptin). Additionally, there has been considerable interest in determining whether insulin resistance (IR) and diabetes are associated with one or more defects in this incretin axis, as well as whether these defects contribute to the development of type 2 diabetes or arise as a consequence of hyperglycemia or other metabolic manifestations of diabetes itself.

Several studies have shown an increased prevalence of IR in patients with rheumatoid arthritis (RA) [5–7], a finding potentially associated with the degree of RA disease activity [8]. It is thought that low-grade inflammation may contribute to its development [9]. This is supported by the fact that IR in patients with RA has been found to directly correlate with levels of interleukin 6, tumor necrosis factor (TNF)-α, and C-reactive protein (CRP) [10]. In addition, anti-TNF-α therapy has been shown to improve insulin sensitivity and reduce IR in RA [11]. However, the precise mechanism linking systemic inflammation with IR in RA remains elusive. In the present study, we sought to determine whether the incretin-insulin axis and incretin effect are impaired in patients with RA, as well as if they are related to the IR found in these patients.

## Methods

### Study participants

We conducted a cross-sectional study that included 361 nondiabetic individuals. Of these, 151 were nondiabetic patients with RA and 210 were sex-matched control subjects. All patients with RA were aged 18 years or older and fulfilled the 2010 American College of Rheumatology/European League Against Rheumatism classification criteria for RA [12]. They had been diagnosed by rheumatologists and were periodically followed at rheumatology outpatient clinics. For the purpose of inclusion in the present study, RA disease duration was required to be ≥ 1 year. Although anti-TNF-α treatment has been associated with changes in IR [5, 13–15], patients with RA undergoing TNF-α antagonist therapy were not excluded in the present study. The control group consisted of patients recruited from the Spanish Camargo Cohort [16, 17]. This cohort was set up between February 2006 and February 2011, and individuals included in this cohort have been followed ever since. The aim of using this cohort was to evaluate the prevalence and incidence of metabolic bone diseases and mineral metabolism disorders. Control subjects included in the present study were subjects without diabetes.

Patients and control subjects with diabetes mellitus were not included in the study. Therefore, none of the patients or control subjects were receiving glucose-lowering drugs or insulin therapy. All patients and control subjects had a glycemia < 7 mmol/L. Patients and control subjects were excluded if they had a history of cardiovascular events that included myocardial infarction, angina, stroke, or peripheral arteriopathy; a glomerular filtration rate < 60 ml/minute/1.73 m^2^; history of cancer; or any other chronic disease or evidence of infection. None of the control subjects were receiving glucocorticoid treatment. However, because prednisone is often used in the management of RA, patients taking this drug or an equivalent dose ≤ 10 mg/day were not excluded. The study protocol was approved by the institutional review committee at Hospital Universitario de Canarias and Hospital Universitario Marqués de Valdecilla (both in Spain), and all subjects provided written informed consent.

### Data collection

Surveys of patients with RA and control subjects were performed in the same way. Subjects completed a cardiovascular risk factor and medication use questionnaire and underwent a physical examination to determine their anthropometrics and blood pressure. Medical records were reviewed to ascertain specific diagnoses and medications. Waist circumference was measured at the smallest circumference point between the rib cage and the iliac crest while the subject was in a standing position. The hip circumference was measured at the widest circumference point between the waist and thighs. The waist-to-hip ratio also was estimated. Hypertension was defined as a systolic or diastolic blood pressure higher than 140 or 90 mmHg, respectively. Dyslipidemia was defined as one of the following metrics being present: total cholesterol > 200 mg/dl, triglyceride > 150 mg/dl, high-density lipoprotein (HDL) cholesterol < 40 mg/dl in men or < 50 mg/dl in women, or low-density lipoprotein (LDL) cholesterol > 130 mg/dl. In patients with RA, disease activity was measured using the Disease Activity Score in 28 joints (DAS28) [18], and disease disability was determined using the Health Assessment Questionnaire [19]. Clinical Disease Activity Index (CDAI) [20] and Simplified Disease Activity Index (SDAI) [21] scores for RA disease activity were obtained as previously described.

### Assessments

The homeostatic model assessment (HOMA) method was performed to determine IR; specifically, in this study, we used HOMA2: the updated computer HOMA model [22, 23]. Briefly, this method consists of a structural computer model of the glucose-insulin feedback system in a homeostatic (overnight-fasted) state. The model is composed of a number of nonlinear empirical equations (and precludes an exact algebraic solution) that describe the functions of organs and tissues involved in glucose regulation. This model can be used to determine insulin sensitivity (%S) and β-cell function (%B) from paired fasting plasma glucose and specific insulin or from C-peptide concentrations across a range of 1–2200 pmol/L for insulin and 1–25 mmol/L for glucose. In our study, we used C-peptide to calculate β-cell function because the former is a marker of secretion. In addition, we used insulin data to calculate %S (because HOMA-%S is derived from glucose disposal as a function of insulin concentration). This computer model provides an insulin sensitivity value expressed as HOMA2-%S (where 100% is normal). HOMA2-IR (IR index) is simply the reciprocal of %S.

Insulin (ARCHITECT i2000; Abbott Diagnostics, Abbott Park, IL, USA) and C-peptide (IMMULITE 2000; Siemens Healthcare, Erlangen, Germany) were determined using chemiluminescent immunometric assays. GLP-1 and GIP were assessed using an enzyme-linked immunosorbent assay (ELISA) (Phoenix Pharmaceuticals, Burlingame, CA, USA). The assay sensitivity (minimum detectable concentration) was 0.11 ng/ml for GLP-1 and 0.47 ng/ml for GIP. These two ELISAs do not cross-react with human insulin, and the presence of insulin in serum does not interfere with the assay results. The kits also have no cross-reactivity with the major species of proinsulin metabolites. The GLP-1 assay does not have cross-reactivity with human GLP-2 or human glucagon. Similarly, the GIP kit does not cross-react with human amylin. Precision was estimated for GLP-1 as interassay 3.79–3.85 and intra-assay 3.81%, for GIP as interassay 3.7–5.06 and intra-assay 4.40%. Serum levels of soluble CD26/DPP-4 were measured through ELISA (R&D Systems, Inc., Minneapolis, MN, USA). The intra-assay and interassay coefficients of variation were 4.2% and 8.1%, respectively. Standard techniques were used to measure plasma glucose, CRP, the Westergren erythrocyte sedimentation rate (ESR), and serum lipids. Blood collected from all the participants by means of venipuncture was stored at 4 °C for < 4 h and then centrifuged, and subsequently serum/plasma was removed and stored at −80 °C.

### Meal test

Ten nondiabetic patients with RA (mean age 45 ± 8 years, body mass index [BMI] 22.6 ± 4.1 kg/m^2^) and ten control subjects (mean age 46 ± 10 years, BMI 26.6 ± 5.2 kg/m^2^) were tested for postprandial levels of glucose, insulin, C-peptide, GIP, and GLP-1 after a meal test. For the purpose of this study, both patients and control subjects must have had a BMI < 30 kg/m^2^. In addition, patients with RA were selected if disease activity was not considered to be in remission (DAS28 ≥ 2.6). In order to avoid the confusing effect it could have, none of the patients with RA were on glucocorticoid therapy. Additional file 1: Table S1 describes the demographic and disease-related characteristics of these 20 subjects in whom the meal test was performed. The test meal consisted of 50 g of white bread, 50 g of black bread, 10 g of butter, 40 g of cheese, 20 g of jam, and 200 ml of milk (34% fat, 47% carbohydrate, and 19% protein), comprising a total of 566 kcal (2370 kJ), and the meal was consumed within 15 minutes. Venous blood was drawn 10 minutes before and 30, 60, 90, 120, 150, 180, 210, and 240 minutes after ingestion of the meal. Blood samples were placed in tubes that were immediately cooled on ice and centrifuged within 20 minutes at 4 °C, and plasma was stored at −20 °C until prompt analysis.

### Statistical analysis

Demographic and clinical characteristics were compared between patients with RA and control subjects using the chi-square test for categorical variables or Student’s *t* test for continuous variables (with data described as mean ± SD). For noncontinuous variables, either the Mann-Whitney *U* test was performed or logarithmic transformation was done, and data were expressed as median and IQR. Binary variables included in Additional file 1: Table S1 were compared using Fisher’s exact test. Differences in glucose homeostasis metabolism molecules and HOMA indexes were studied using three different linear multivariable regression models: a univariate unadjusted model; a second model adjusted for those variable with a *p* value < 0.20 in the differences between patients and control subjects (age, sex, waist circumference, dyslipidemia, statins, antihypertensive treatment, and CRP and cholesterol levels); and a third model with the same variables, though with the addition of glucocorticoid intake as a binary variable. The association of incretins and DPP-4 with HOMA2 indexes was assessed with multivariable regression analysis performed with the predictive data of the adjusted model (for age, sex, waist circumference, dyslipidemia, statins, antihypertensive treatment, CRP and cholesterol levels, and glucocorticoid intake). Differences between control subjects and patients in the β coefficients of the relationship between incretins (independent variable) and HOMA2 IR indexes (dependent variable) were assessed by adding incretins × RA as an interaction factor into the linear regression models. For the meal test, early response from baseline to minute 60, late response from minute 60 to minute 240, maximum response (expressed in the molecule units as median and IQR) and minutes to maximum response were defined. Differences between AUC in the meal test were calculated using the DeLong method [24]. For all analyses, we used a 5% two-sided significance level, and all analyses were performed using IBM SPSS Statistics version 21 software (IBM, Armonk, NY, USA) and Stata version 13/SE software (StataCorp, College Station, TX, USA). A *p* value < 0.05 was considered statistically significant.

## Results

### Demographic, analytical, and disease-related data

A total of 361 nondiabetic participants comprising 151 patients with RA, and 210 control subjects with mean ± SD ages of 53 ± 11 years and 58 ± 9 years (*p* = 0.00), respectively, were included in this study. The demographic and disease-related characteristics of the participants are shown in Table 1. There were no differences between patients and control subjects regarding BMI, although waist circumference was found to be higher in patients than in control subjects (92 ± 14 vs. 96 ± 13 cm, *p* = 0.015). The frequency of hypertension was not different between patients and control subjects. This was not the case for the lipid profile. In this regard, patients with RA had lower levels of total cholesterol (206 ± 36 vs. 219 ± 37 mg/dl, *p* = 0.000), LDL cholesterol (121 ± 31 vs. 135 ± 34 mg/dl, *p* = 0.000), HDL cholesterol (56 ± 15 vs. 63 ± 18 mg/dl, *p* = 0.000), and apolipoprotein A1 (170 ± 28 vs. 191 ± 35 mg/dl, *p* = 0.000). In contrast, triglycerides, lipoprotein A, and apolipoprotein B were found to be higher in patients with RA.

**Table 1:** Demographics, analytical data, and disease-related characteristics of patients with rheumatoid arthritis and control subjects

	Control subjects (*n* = 210)	Patients with RA (*n* = 151)	*p* Value
Age, years	58 ± 9	53 ± 11	**0.000**
Female sex, *n* (%)	148 (70)	120 (79)	0.052
Body mass index, kg/m^2^	28 ± 5	28 ± 5	0.78
Waist circumference, cm	92 ± 14	96 ± 13	**0.015**
Cardiovascular risk factors, *n* (%)			
Smoking	42 (20)	26 (17)	0.51
Hypertension	58 (27)	45 (30)	0.65
Dyslipidemia	33 (15)	54 (36)	**0.000**
Diabetes	0 (0)	0 (0)	–
Medication			
Statins	14 (6)	43 (28)	**0.000**
Antihypertensive treatment	35 (16)	45 (30)	**0.003**
Analytical data			
CRP, mg/dl	1.0 (1.0–3.0)	3.2 (1.5–5.8)	**0.000**
ESR, mm/h	10 ± 8	34 ± 22	**0.000**
Triglycerides, mg/dl	104 ± 50	144 ± 92	**0.000**
HDL cholesterol, mg/dl	63 ± 18	56 ± 15	**0.000**
LDL cholesterol, mg/dl	135 ± 34	121 ± 31	**0.000**
Total cholesterol, mg/dl	219 ± 37	206 ± 36	**0.001**
Lipoprotein A, mg/dl	16 (9–39)	35 (11–121)	**0.000**
Apolipoprotein A1, mg/dl	191 ± 35	170 ± 28	**0.000**
Apolipoprotein B, mg/dl	102 ± 24	110 ± 63	0.11
ApoB/ApoA ratio	0.55 ± 0.16	0.66 ± 0.30	**0.000**
Atherogenic index	3.7 ± 4.0	4.0 ± 3.6	0.10
Disease-related data			
Disease duration, years		6.6 (3.3–13.9)	
ACPA, *n* (%)		89 (59)	
Rheumatoid factor, *n* (%)		109 (72)	
Erosions, *n* (%)		55 (36)	
Extra-articular manifestations, *n* (%)		16 (11)	
DAS28-ESR		3.7 ± 1.2	
DAS28-CRP		2.9 ± 1.0	
SDAI		15 (8–21)	
CDAI		9 (5–16)	
HAQ		0.630 (0.380–1.130)	
Current prednisone, *n* (%)		57 (38)	
Prednisone current doses, mg/day		5 (5–6)	
NSAIDs, *n* (%)		69 (46)	
DMARDs, *n* (%)		129 (85)	
Methotrexate, *n* (%)		113 (75)	
Biologic drugs, *n* (%)		35 (23)	
Anti-TNF-α drugs, *n* (%)		20 (13)	

*Abbreviations: ACPA* Anticitrullinated peptide/protein antibody, *Apo* Apolipoprotein, *BMI* Body mass index, *CDAI* Clinical Disease Activity Index, *CRP* C-reactive protein, *DAS28* Disease Activity Score in 28 joints, *DMARD* Disease-modifying antirheumatic drug, *ESR* Erythrocyte sedimentation rate, *HAQ* Health Assessment Questionnaire, *HDL* High-density lipoprotein, *LDL* Low-density lipoprotein, *NSAID* Nonsteroidal anti-inflammatory drug, *RA* Rheumatoid arthritis, *SDAI* Simplified Disease Activity Index, *TNF-α* Tumor necrosis factor-α

Current prednisone doses pertain to prednisone users only. Data are expressed as mean ± SD or median (IQR). Dichotomous variables are expressed as count and percent. *p*<0.05 are depicted in bold

As expected, the assessment of ESR and CRP values revealed statistically significant higher levels in patients with RA. Patients from our series had moderate active disease as shown by DAS28 (3.7 ± 1.2), and 50 (38%) of them were on prednisone (median dose 5 [IQR 5–6] mg/day). Disease duration was 6.6 (IQR 3.3–13.9) years, and 59% and 72% were positive for anticitrullinated protein antibodies and rheumatoid factor, respectively. In addition, whereas 85% of the patients were on disease-modifying antirheumatic drugs, 13% were on anti TNF-α treatment and 23% were receiving biologic therapy.

### Differences in carbohydrate metabolism molecules, incretins, and insulin resistance indexes between patients with RA and control subjects

HOMA2-IR indexes, whether calculated with insulin or C-peptide, were different between patients and control subjects (Table 2). In this sense, HOMA2-S% was lower in patients with RA than in control subjects after adjusting for traditional IR-related factors and prednisone intake (105 ± 53 vs. 108 ± 75, *p* = 0.006). Similarly, HOMA2-IR was found to be higher in patients with RA than in control subjects after multivariable analysis (1.27 ± 0.82 vs. 1.65 ± 1.69, *p* = 0.054). In contrast, HOMA2-%B was higher in patients with RA in the univariate analysis. However, the difference was lost after adjustment for covariables (*p* = 0.14).

**Table 2:** Linear regression analysis of differences in carbohydrate metabolism molecules, incretin hormones, and insulin resistance indexes between patients with rheumatoid arthritis and control subjects

			Univariate model	Adjusted model		Adjusted model + GC	
	Control subjects (*n* = 210)	Patients with RA (*n* = 151)	*p* Value	β Coefficient (95% CI)	*p* Value	β Coefficient (95% CI)	*p* Value
Glucose, mg/dl	90 ± 10	88 ± 19	0.21	3 (−1 to 7)	0.37	1 (−4 to 5)	0.71
Insulin, U/ml	9.8 ± 6.5	13.0 ± 13.4	**0.007**	3.0 (0.2 to 5.8)	**0.037**	3.2 (0.1 to 6.4)	**0.046**
C-peptide, ng/ml	1.53 ± 0.77	3.37 ± 2.94	**0.000**	2.13 (1.56 to 2.69)	**0.000**	1.98 (1.35 to 2.61)	**0.000**
GLP-1, ng/ml	0.49 ± 1.28	0.71 ± 0.22	0.06	0.31 (0.25 to 0.38)	**0.000**	0.32 (0.25 to 0.39)	**0.000**
GIP, ng/ml	0.37 ± 0.40	1.78 ± 0.51	**0.000**	1.35 (1.19 to 1.51)	**0.000**	1.33 (1.15 to 1.51)	**0.000**
DPP-4, ng/ml	811 ± 459	696 ± 301	**0.007**	−112 (−213 to −10)	**0.032**	−85 (−199 to 30)	0.15
HOMA2 insulin							
HOMA2-IR	1.27 ± 0.82	1.65 ± 1.69	**0.011**	0.38 (0.03 to 0.74)	**0.036**	0.39 (−0.01 to 0.79)	0.054
HOMA2-%S	108 ± 75	105 ± 53	0.65	−19 (3 to −35)	**0.019**	−25 (−7 to −42)	**0.006**
HOMA2-%B	111 ± 45	134 ± 69	**0.000**	8 (−8 to 23)	0.33	13 (−4 to 31)	0.14
HOMA2 C-peptide							
HOMA2-IR	1.13 ± 0.58	2.49 ± 2.35	**0.000**	1.63 (−1.18 to 2.09)	**0.000**	1.51 (1.00 to 2.02)	**0.000**
HOMA2-%S	115 ± 75	67 ± 41	**0.000**	−51 (−68 to −34)	**0.000**	−45 (−64 to −28)	**0.000**
HOMA2-%B	104 ± 36	180 ± 82	**0.000**	71 (55 to 88)	**0.000**	74 (55 to 93)	**0.000**

*Abbreviations: DPP-4* Dipeptidyl peptidase 4, *GC* Glucocorticoid, *GIP* Gastric inhibitory polypeptide, *GLP-1* Glucagon-like peptide 1, *HOMA* Homeostatic model assessment, *HOMA2-%B* Homeostatic model assessment of β-cell production calculated with C-peptide, *HOMA2-IR* Homeostatic model assessment calculated with insulin, *RA* Rheumatoid arthritis

Adjusted model for age, sex, waist circumference, dyslipidemia, statins, antihypertensive treatment, and C-reactive protein and cholesterol levels. Analytical data represent unadjusted values: β coefficient (95% CI). *p*<0.05 are depicted in bold

When HOMA2 indexes were constructed with C-peptide, the differences between patients and control subjects were found to be stronger. In this regard, all comparisons disclosed higher HOMA2-IR and HOMA2-%B indexes and lower HOMA2-%S in patients with RA even after multivariable analysis (Table 2).

Whereas glucose serum levels were not different between control subjects and patients, insulin (9.8 ± 6.5 vs. 13.0 ± 13.4 U/ml, *p* = 0.007) and C-peptide serum levels (1.53 ± 0.77 vs. 3.37 ± 2.94 ng/ml, *p* = 0.000) were found to be upregulated in patients with RA. These differences were maintained after multivariable adjustment including glucocorticoid intake. Similarly, GLP-1 (0.49 ± 1.28 vs. 0.71 ± 0.22 ng/ml, *p* = 0.06 in univariate analysis) and GIP (0.37 ± 0.40 vs. 1.78 ± 0.51 ng/ml, *p* = 0.000) were higher in patients with RA than in control subjects. These differences were also present after adjusting for factors related to IR and prednisone intake; in the case of GLP-1, it reached statistical significance (*p* = 0.000) after multivariable analysis. In contrast, DPP-4 soluble-form serum levels were found to be significantly lower in patients with RA than in control subjects (811 ± 459 vs. 696 ± 301 ng/ml) in univariate analysis. These differences were out of the range of significance after adjustment (*p* = 0.15) (Table 2).

### Laboratory markers of inflammation, disease-related data, and disease activity relationship vis-à-vis incretins, DPP-4, and HOMA-IR indexes

Regarding inflammatory serum markers, CRP and ESR showed a significant positive correlation with HOMA2-%B and GIP, respectively (β coefficient [95% CI 0.01 [0.00–0.01] ng/ml, *p* = 0.033]. Although disease duration and rheumatoid factor were not related to carbohydrate metabolism molecules, the presence of anticitrullinated peptide/protein antibody was negatively associated with insulin and C-peptide serum levels and HOMA2-%B, but it was positively correlated with DDP-4 levels (β coefficient 157 [95% CI 58–256] ng/ml, *p* = 0.002). Disease activity scores through DAS28-ESR (β coefficient 46 [95% CI 6–87], *p* = 0.026) and CDAI (β coefficient 7.74 [95% CI 1.29–14.20] 0.019) were positively associated with DPP-4 serum levels but not with other molecules or HOMA-IR indexes (Table 3). Furthermore, disease activity stratification did not yield any additional associations.

**Table 3:** Inflammatory markers, disease-related data, and disease activity scores: univariate relationship with insulin and C-peptide serum levels, incretins, dipeptidyl peptidase 4, and homeostatic model assessment calculated with insulin indexes

	β Coefficient (95% CI), *p* value						
	Insulin, U/ml	C-peptide, ng/ml	GLP-1, ng/ml	GIP, ng/ml	DPP-4, ng/ml	HOMA2-IR insulin	HOMA2-%B C-peptide
CRP, mg/dl	0.02 (−0.05 to 0.08), 0.61	0.01 (−0.02 to 0.03), 0.09	0.00 (−0.00 to 0.00), 0.70	0.00 (−0.00 to 0.01), 0.44	−1 (−4 to 2), 0.64	0.00 (−0.01 to 0.01), 0.61	**0.54 (0.16 to 0.96), 0.013**
ESR, mm/h	−0.01 (−0.10 to 0.09), 0.88	0.00 (−0.02 to 0.03), 0.67	0.00 (−0.00 to 0.00), 0.47	**0.01 (0.00 to 0.01), 0.033**	1 (−1 to 3), 0.37	−0.00 (−0.01 to 0.01), 0.90	0.08 (−0.50 to 0.66), 0.79
Disease duration, years	−0.03 (−0.27 to 0.20), 0.78	0.01 (−0.05 to 0.06), 0.82	−0.00 (−0.01 to 0.00), 0.40	0.01 (−0.00 to 0.02), 0.20	2 (−3 to 8), 0.40	−0.00 (−0.03 to 0.03), 0.82	−0.51 (−1.95 to 0.94), 0.49
ACPA	**−4.57 (−9.09 to −0.04), 0.048**	**−1.11 (−2.10 to −0.11), 0.029**	0.02 (−0.07 to 0.10), 0.70	−0.01 (−0.20 to 0.18), 0.91	**157 (58 to 256), 0.002**	−0.57 (−1.14 to 0.01), 0.053	**−32.23 (−60.02 to −4.44), 0.023**
Rheumatoid factor	−1.41 (−6.45 to 3.63), 0.58	−0.10 (−1.21 to 1.01), 0.85	0.04 (−0.06 to 0.13), 0.44	0.10 (−0.10 to 0.31), 0.33	76 (−35 to 188), 0.18	−0.13 (−0.77 to 0.50), 0.68	−18.37 (−49.31 to 12.57), 0.24
DAS28-ESR	−0.49 (−2.32 to 1.35), 0.60	−0.01 (−0.41 to 0.40), 0.98	0.01 (−0.03 to 0.4), 0.65	−0.04 (−0.12 to 0.03), 0.24	**46 (6 to 87), 0.026**	−0.06 (−0.29 to 0.18), 0.64	−0.79 (−12.11 to 10.53), 0.89
DAS28-CRP	−0.22 (−2.43 to 2.00), 0.85	0.08 (−0.41 to 0.57), 0.75	0.01 (−0.03 to 0.05), 0.64	−0.05 (−0.14 to 0.04), 0.30	45 (−5 to 94), 0.075	−0.02 (−0.30 to 0.26), 0.88	3.33 (−10.31 to 16.97), 0.63
SDAI	0.00 (−0.10 to 0.11), 0.96	0.01 (−0.02 to 0.03), 0.61	−0.00 (−0.00 to 0.00), 0.34	0.00 (−0.00 to 0.00), 0.99	1.49 (−0.87 to 3.85), 0.21	0.00 (−0.01 to 0.01), 0.97	0.38 (−0.26 to 1.03), 0.24
CDAI	−0.06 (−0.36 to 0.23), 0.67	−0.00 (−0.07 to 0.06), 0.90	0.00 (−0.00 to 0.01); 0.40	−0.01 (−0.02 to 0.00); 0.075	**7.74 (1.29 to 14.2), 0.019**	−0.01 (−0.05 to 0.03), 0.68	0.02 (−1.78 to 1.82), 0.98
SDAI	−0.01 (−0.11 to 0.10), 0.92	0.00 (−0.02 to 0.03), 0.72	−0.00 (−0.00 to 0.00), 0.28	0.00 (−0.00 to 0.01), 0.84	1.24 (−1.16 to 3.64), 0.31	−0.00 (−0.01 to 0.01), 0.91	0.34 (−0.32 to 0.99), 0.31
CDAI	0.01 (−0.04 to 0.05), 0.73	0.00 (−0.01 to 0.01), 0.41	0.00 (−0.00 to 0.00), 0.35	−0.00 (−0.00 to 0.00), 0.14	**1.27 (0.28 to 2.26), 0.012**	0.00 (−0.01 to 0.01), 0.72	0.12 (−0.16 to 0.39), 0.41
Prednisone intake	2.56 (−0.31 to 5.42), 0.080	**1.69 (1.09 to 2.28), 0.000**	**0.19 (0.11 to 0.27), 0.000**	**0.96 (0.72 to 1.19), 0.000**	**−131 (−244 to −18), 0.023**	0.34 (−0.02 to 0.70), 0.067	**51 (31 to 70), 0.000**
Methotrexate	−9.9 (−44 to 25), 0.57	−0.11 (−0.47 to 0.25), 0.55	0.02 (−0.07 to 0.11), 0.73	0.05 (−0.15 to 0.25), 0.60	36 (−76 to 147), 0.53	−0.19 (−0.82 to 0.44), 0.55	−5 (−36 to 25), 0.73
Anti-TNF-α drugs	28 (−16 to 72), 0.21	0.21 (−0.26 to 0.67), 0.39	−0.10 (−0.21 to 0.02), 0.11	0.09 (−0.16 to 0.34), 0.48	30 (−113 to 173), 0.68	0.54 (−0.27 to 1.34), 0.19	−12 (−51 to 27), 0.55

*Abbreviations: ACPA* Anticitrullinated peptide/protein antibody, *CDAI* Clinical Disease Activity Index, *CRP* C-reactive protein, *DAS28* Disease Activity Score in 28 joints, *DPP-4* Dipeptidyl peptidase 4, *ESR* Erythrocyte sedimentation rate, *GIP* Gastric inhibitory polypeptide, *GLP-1* Glucagon-like peptide 1, *HOMA2-%B* Homeostatic model assessment of β-cell production calculated with C-peptide, *HOMA2-IR* Homeostatic model assessment calculated with insulin, *SDAI* Simplified Disease Activity Index, *TNF-α* Tumor necrosis factor-α. *p*<0.05 are depicted in bold

With regard to RA treatments, neither methotrexate nor anti-TNF-α inhibitors were related to insulin, incretins, DPP-4 serum levels, or IR indexes. In contrast, glucocorticoids were significantly associated with higher levels of insulin, C-peptide, GLP-1, GIP, and HOMA-IR indexes, as well as with lower levels of DPP-4 (Table 3).

### Relation of incretins and DPP-4 to IR indexes

DPP-4 serum levels showed a correlation with IR (HOMA2-IR) and β-cell secretion (HOMA2-%B-C-peptide) in both patients and control subjects after multivariate regression analysis. In both cases, they had a negative and statistically significant correlation with both indexes. A β-coefficient comparison revealed no differences, showing that the relationship of DPP-4 to these indexes, whether in patients or in control subjects, did not differ (Table 4).

**Table 4:** Multivariable analysis of relationship of incretins and dipeptidyl peptidase 4 with insulin resistance indexes

	β Coefficient (95% CI)			β Coefficient (95% CI)		
	HOMA-IR	*p* Value	*p* Value*	HOMA2%B-C-peptide	*p* Value	*p* Value*
GLP-1, ng/ml						
Patients with RA	0.00 (−0.93 to 0.93)	0.99	0.068	**155 (105 to 205)**	**0.000**	**0.003**
Control subjects	**−1.34 (−2.46 to −0.23)**	**0.018**		37 (−22 to 96)	0.22	
GIP, ng/ml						
Patients with RA	**0.32 (0.10 to 0.53)**	**0.004**	0.42	**59 (50 to 68)**	**0.000**	0.29
Control subjects	0.14 (−0.22 to 0.51)	0.44		**49 (31 to 66)**	**0.000**	
DPP-4, ng/ml						
Patients with RA	**−0.002 (−0.003 to −0.002)**	**0.000**	0.39	**−0.12 (−0.16 to −0.09)**	**0.000**	0.71
Control subjects	**−0.003 (−0.003 to −0.002)**	**0.000**		**−0.13 (−0.18 to 0.09)**	**0.000**	

*Abbreviations: DPP-4* Dipeptidyl peptidase 4, *GIP* Gastric inhibitory polypeptide, *GLP-1* Glucagon-like peptide 1, *HOMA2-%B* Homeostatic model assessment of β-cell production calculated with C-peptide, *HOMA2-IR* Homeostatic model assessment calculated with insulin, *RA* Rheumatoid arthritis

All analyses were adjusted for age, sex, waist circumference, dyslipidemia, statins, antihypertensive treatment, and CPR and cholesterol levels. GLP-1, GIP and DPP-4 are considered independent variables. β Coefficients of the relation of GLP-1, GIP and DPP-4 (independent variables) with HOMA2 indexes (dependent variables) are disclosed. *p* Values are the linear regression adjusted *p* values of these associations. p* Value refers to the statistical significance of the interaction factor when adding GLP-1 × RA, GIP × RA, and DPP-4 × RA as such interaction factors to the model. *p*<0.05 are depicted in bold

GIP had a statistically significant association with HOMA2-%B-C-peptide in both patients and control subjects, which did not differ between the two populations (interaction *p* = 0.29). Otherwise, GIP was found to be related to HOMA2-IR in patients with RA but not in control subjects, although the interaction factor in this case was not significant. Regarding GLP-1, its relationship to HOMA-IR (interaction *p* = 0.068) and HOMA2%-C-peptide (interaction *p* = 0.003) was different between patients and control subjects. In fact, although the relationship of GLP-1 to HOMA-IR was found to be negative in control subjects (β coefficient −1.34 [95% CI −2.46 to −0.23], *p* = 0.018), its correlation with HOMA2-%B-C-peptide was found to be positive and statistically significant in patients with RA (β coefficient 155 [95% CI 105–205], *p* = 0.000) (Table 4).

### Meal test

Additional file 1: Table S1 shows the characteristics of patients and control subjects who underwent the meal test. Traditional cardiovascular risk factors, use of medications, and laboratory data did not differ between patients and control subjects. Only waist circumference was found to be higher in patients with RA (75.3 ± 11.8 vs. 95.3 ± 10.0 cm, *p* = 0.028). Fasting GIP serum levels were also higher in patients with RA than in control subjects (0.93 ± 0.14 vs. 1.14 ± 0.18 ng/ml, *p* = 0.029).

**Fig. 1:**
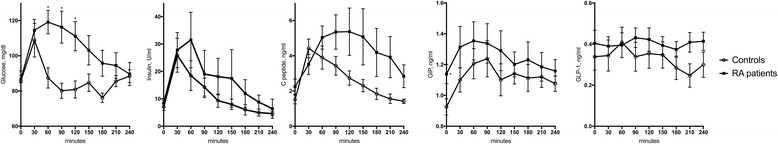
Meal test curves of glucose, insulin, C-peptide, gastric inhibitory polypeptide, and glucagon-like peptide 1 concentrations in patients with rheumatoid arthritis and control subjects

The AUC of glucose response after the meal test was found to be higher in patients with RA compared with control subjects (691 ± 78 vs. 843 ± 114, *p* = 0.006). Late-response AUC in glucose response was also higher in patients with RA, although statistical significance was not reached (*p* = 0.054). Similarly, C-peptide minutes until maximal response was higher in patients with RA compared with control subjects (30 [30-60] vs. 75 [60-105] minutes, *p* = 0.029). Although an insulin AUC comparison between patients and control subjects showed no differences, visually AUC was higher in patients with RA and slower than in control subjects. Moreover, GIP response had a flatter curve in patients with RA, although statistical significance was not reached in this case (Table 5 and Fig. 1).

**Table 5:** Glucose, insulin, C peptide, GIP and GLP-1 concentrations after meal test in RA patients and controls

		Glucose	p	Insulin	p	C peptide	p	GIP	p	GLP-1	p
Maximum response	Controls	102 (96-141)	0.11	27.2 (11.2-44.8)	0.49	4.98 (2.99-6.03)	0.20	1.49 (1.22-2.08)	0.73	0.59 (0.24-0.61)	0.73
	RA patients	134 (127-138)		26.8 (12.8-56.0)		5.85 (3.98-7.96)		1.48 (1.37-1.81)		0.48 (0.45-0.62)	
Minutes to max. response	Controls	30 (30-240)	0.39	30 (30-60)	0.36	30 (30-60)	**0.029**	150 (60-210)	0.08	60 (60-120)	0.34
	RA patients	75 (45-90)		45 (30-105)		75 (60-105)		45 (15-135)		45 (30-105)	
AUC	Controls	691 ± 78	**0.006**	92 ± 52	0.87	21 ± 7	0.23	9 ± 2	0.69	3 ± 1	0.34
	RA patients	843 ± 114		105 ± 84		35 ± 21		10 ± 2		3 ± 1	
Early response AUC	Controls	195 ± 39	0.34	39 ± 23	0.99	7 ± 2	0.87	2.2 ± 0.3	0.28	0.7 ± 0.4	0.78
	RA patients	217 ± 21		38 ± 29		7 ± 2		2.6 ± 0.6		0.8 ± 0.3	
Late response AUC	Controls	411 ± 44	**0.054**	33 ± 24	0.37	11 ± 3	0.12	6.0 ± 1.0	0.61	1.7 ± 0.8	0.40
	RA patients	507 ± 99		59 ± 48		23 ± 17		6.2 ± 1.2		2.0 ± 0.6	

Data expressed as median (interquartile range)

AUC are depicted as average ± standard deviation

Early response refers to the AUC from baseline to minute 60

Late response refers to the AUC from minute 60 to minute 240

Maximum response is expressed in the molecules units median and interquartile range

Minutes to maximum response is expressed in median and interquartile range

GLP-1: Glucagon-Like Peptide 1; GIP: gastric inhibitory polypeptide

## Discussion

The present study shows, for the first time, to our knowledge, the expression of incretins in patients with RA. According to our findings, incretins and DPP-4 differ in patients with RA and control subjects. This is related to disease activity and glucocorticoid intake. We also observed that insulin, C-peptide levels, and HOMA-IR indexes are independently elevated in patients with RA compared with control subjects.

We have previously demonstrated that β-cell function is impaired in patients with RA because of the elevation in serum levels of split and intact forms of proinsulin [7]. In keeping with former studies [8, 25, 26], our data confirmed the association between IR and RA. In this context, Chung et al. studied IR in 104 patients with RA and 124 patients with systemic lupus erythematosus (SLE). The former had a higher IR index than those with SLE, and IR showed a positive correlation with the levels of proinflammatory cytokines interleukin-6, TNF-α, and CRP [27]. Severe IR has been also found to be present in patients with early untreated RA [28].

Type 2 diabetes mellitus was thought to be characterized by a severely impaired or absent GIP insulinotropic effect that most likely resulted in worsening insulin secretion. However, current analyses have revealed that type 2 diabetes seems unlikely to result from deficient incretin secretion [29]. On the basis of results obtained during the course of both oral glucose tolerance testing and meal testing, GIP secretion and fasting levels actually seem to increase in both the impaired and diabetic state [30]. In our study, fasting incretin serum levels were higher in patients with RA than in control subjects. This increase in incretin levels was in keeping with the upregulation of insulin and C-peptide. In contrast, DPP-4 was found to be downregulated in patients with RA. We believe that DPP-4 downregulation is consistent with the increase in incretins due to the accepted opposite relationship of incretins and DPP-4. Interestingly, DPP-4 was also found to be positively related to disease activity through DAS28-ESR and CDAI scores.

Previous reports have shown decreased enzymatic activity and low DPP-4 serum levels in patients with RA compared with those of healthy control subjects [31, 32]. However, an increase in the number of peripheral T lymphocytes expressing DPP-4 has been reported in patients with active RA [33]. This apparent contradictory result may explain the positive correlation of DPP-4 with disease activity observed in our patients with RA. Of note, a recent study involving 50 patients with RA revealed a decrease in DPP-4 serum activity but not in DPP-4/CD26 expression [34]. In another study involving 27 patients with RA, there was also an elevation in blood plasma DPP-4 but a decrease of DPP-4/CD26 in peripheral blood mononuclear cells after clinical improvement following treatment [35]. Taking these observations into account, we feel that the number of peripheral T lymphocytes expressing DPP-4/CD26 is higher in the blood of patients with active RA. In contrast, however, the enzymatic activity and serum levels of DPP-4/CD26 may be lower in the sera of patients with RA than in those of healthy control subjects.

Interestingly, prednisone intake was associated with higher levels of incretins and lower levels of DPP-4. To the best of our knowledge, there are no studies focused on the effects of glucocorticoids over incretins or DPP-4 in chronic diseases. We believe that the mechanism by which glucocorticoids impair incretin may be similar to that underlying increases in insulin and C-peptide: The glucose homeostasis disruption and IR state that they induce probably lead to a secondary and compensatory elevation in incretins, as occurs with insulin and C-peptide.

We also assessed whether the relationships of incretins and DPP-4 with insulin resistance (HOMA2-IR) and β-cell function (HOMA2-%B-C-peptide) in patients with RA differed from those in control subjects. Interestingly, we observed a different relationship of HOMA indexes with GLP-1 but not with GIP or DPP-4. It is known that GIP does not modulate glucose-dependent insulin secretion in type 2 diabetes, even at supraphysiological (pharmacological) plasma levels. Therefore, GIP incompetence is detrimental to β-cell function, especially after eating. GLP-1 remains insulinotropic in type 2 diabetes, and this fact has led to the development of compounds that activate the GLP-1 receptor with a view to improving insulin secretion [30]. In our study, we found that GLP-1 was negatively related to HOMA-IR in control subjects but not in patients with RA. In contrast, GLP-1 was positively related to β-cell function (HOMA2-%B-C-peptide) in patients with RA but not in control subjects. We do not have an explanation for this finding. We believe that although the association of GLP-1 with IR can be lost in RA, it could still remain as an insulinotropic agent in terms of enhancing higher β-cell secretion in these patients.

We also focused on studying incretins in a nonfasting (postprandial) state. To this end, we performed a meal test in nonobese control subjects and patients who were not taking glucocorticoids and who had moderate or high disease activity. Reports regarding meal tests in other populations, such as subjects with diabetes, used similar numbers of individuals to those of our study [36] because the meal test is technically complex and requires a well-trained team. We feel that our results may indicate that the meal test is different in patients with RA when compared with control subjects and that the expression of incretins after this meal test is altered in patients with RA. In this regard, in patients with RA, glucose, insulin, and GLP-1 curves were abnormal compared with those of control subjects. With respect to the trends observed in the meal test, we think that the assessment of a larger series of patients and control subjects could have led to stronger results in terms of statistical significance. Nevertheless, to the best of our knowledge, such findings regarding the meal test in patients with RA have not previously been reported in the literature.

## Conclusions

Our study, which, to our knowledge, constitutes the first assessment of the incretin-insulin axis and the incretin effect in RA, shows that these molecules (metabolic hormones) are impaired in patients with RA. The presence of this impairment reinforces the concept that the disease itself, probably by means of the effect of inflammation, leads to an IR state. Our results demonstrate the existence of a mechanism linking inflammation with IR that warrants further studies.

## Additional file

Additional file 1: Table S1. Demographics, analytical data, and disease-related characteristics of patients with RA and control subjects who underwent the meal test. (DOC 64 kb)

## Abbreviations

ACPA: Anticitrullinated peptide/protein antibody
Apo: Apolipoprotein
BMI: Body mass index
CDAI: Clinical Disease Activity Index
CRP: C-reactive protein
DAS28: Disease Activity Score in 28 joints
DMARD: Disease-modifying antirheumatic drug
DPP-4: Dipeptidyl peptidase 4
ELISA: Enzyme-linked immunosorbent assay
ESR: Erythrocyte sedimentation rate
GC: Glucocorticoid
GIP: Gastric inhibitory polypeptide
GLP-1: Glucagon-like peptide 1
HAQ: Health Assessment Questionnaire
HDL: High-density lipoprotein
HOMA: Homeostatic model assessment
HOMA2-%B: Homeostatic model assessment of β-cell production calculated with C-peptide
HOMA2-IR: Homeostatic model assessment calculated with insulin
IR: Insulin resistance
LDL: Low-density lipoprotein
NSAID: Nonsteroidal anti-inflammatory drug
RA: Rheumatoid arthritis
SDAI: Simplified Disease Activity Index
SLE: Systemic lupus erythematosus
TNF-α: Tumor necrosis factor-α
