# Supplementary material for: Incretins in patients with rheumatoid arthritis
Source: Arthritis Res Ther. 2017 Oct 17;19:229. doi: 10.1186/s13075-017-1431-9 (PMC5645916; doi:10.1186/s13075-017-1431-9)
Supplement: Additional file 1: Table S1. — Demographics, analytical data, and disease-related characteristics of patients with RA and control subjects who underwent the meal test. (DOC 64 kb) [file 13075_2017_1431_MOESM1_ESM.doc]

| **Additional file 1: Table S1. Demographics, analytical data and disease-related** | | | | |
| --- | --- | --- | --- | --- |
| **characteristics of RA patients and controls who underwent the meal test** | | | | |
|  |  | Controls | RA |  |
|  |  | (n=10 ) | (n=10) | p |
|  | Age, years | 45 ± 8 | 46 ± 10 | 0.99 |
|  | Female, n(%) | 9 (90) | 9 (90) | 0.99 |
|  | Body mass index, kg/m2 | 22.6 ± 4.1 | 26.6 ± 5.2 | 0.22 |
|  | Waist circumference, cm | 75.3 ± 11.8 | 95.3 ± 10.0 | **0.028** |
| Cardiovascular risk factors | |  |  |  |
|  | Smoking, n(%) | 0 (0) | 1 (10) | 0.99 |
|  | Hypertension, n(%) | 0 (0) | 4 (40) | 0.087 |
|  | Dyslipidemia, n(%) | 0 (0) | 3 (30) | 0.21 |
|  | Diabetes, n(%) | 0 (0) | 0 (0) | - |
| Medication | |  |  |  |
|  | Statins | 0 (0) | 2 (20) | 0.47 |
|  | Antihypertensive treatment | 0 (0) | 30 (30) | 0.21 |
| Analytical data | |  |  |  |
|  | CRP, mg/dL | 1.6 ± 0.9 | 1.8 (0.8-12.6) | 0.54 |
|  | Triglycerides, mg/dL | 89 ± 52 | 162 ± 76 | 0.082 |
|  | HDL-C, mg/dL | 61 ± 16 | 54 ± 17 | 0.79 |
|  | LDL-C, mg/dL | 141 ± 35 | 103 ± 36 | 0.082 |
|  | Total Cholesterol, mg/dL | 220 ± 40 | 190 ± 42 | 0.25 |
|  | Lipoprotein A, mg/dL | 115 (33-187) | 10 (4-24) | 0.052 |
|  | Apolipoprotein A1, mg/dL | 155 ± 24 | 156 ± 30 | 0.93 |
|  | Apolipoprotein B, mg/dL | 110 ± 30 | 94 ± 21 | 0.54 |
|  | ApoB : ApoA ratio | 0.73 ± 0.29 | 0.62 ± 0.20 | 0.66 |
|  | Atherogenix index | 3.84 ± 1.50 | 3.69 ± 0.97 | 0.93 |
| Glucose homeostasis metabolism | |  |  |  |
|  | Glucose, mg/dL | 86 ± 15 | 87 ± 6 | 0.40 |
|  | Insulin, U/mL | 7.1 ± 3.4 | 8.4 ± 2.7 | 0.34 |
|  | C-peptide, ng/mL | 1.51 ± 0.65 | 2.25 ± 1,21 | 0.15 |
|  | Amylin, ng/mL | 1.16 ± 0.54 | 2.50 ± 2.55 | 0.23 |
|  | GLP-1, ng/mL | 0.34 ± 0.17 | 0.40 ± 0.18 | 0.78 |
|  | GIP, ng/mL | 0.93 ± 0.14 | 1.14 ± 0.18 | **0.029** |
| Disease related data | |  |  |  |
|  | Disease duration, years |  | 12 ± 5 |  |
|  | ACPA, n(%) |  | 9 (90) |  |
|  | Rheumatoid factor, n(%) |  | 9 (90) |  |
|  | Erosions, n(%) |  | 10 (10) |  |
|  | Extra articular manifestations, n(%) |  | 2 (20) |  |
|  | DAS 28-ESR |  | 3.27 ± 1.51 |  |
|  | DAS 28-CRP |  | 3.05 ± 1.03 |  |
|  | SDAI |  | 15 ± 9 |  |
|  | CDAI |  | 72 ± 54 |  |
|  | HAQ |  | 0.750 (0.250-1.313) |  |
|  | Current prednisone, n(%) |  | 0 (0) |  |
|  | NSAIDs. n(%) |  | 4 (40) |  |
|  | DMARDs, n(%) |  | 10 (100) |  |
|  | Methotrexate, n(%) |  | 10 (100) |  |
| Data expressed as mean (± standard deviation) or median (interquartile range). | | | | |
| Dichotomous variables are expressed as n and percentage. DAS28: Disease Activity Score. | | | | |
| ACPA: Anti-citrullinated peptide/protein antibody; DMARD: Disease-modifying Antirheumatic Drug | | | | |
| ESR: erythrocyte sedimentation rate; CRP: C reactive protein; HAQ: Health Assessment Questionnaire. | | | | |
| SDAI: Simple Disease Activity Index; CDAI: Clinical Disease Activity Index | | | | |
| HDL-C: high-density cholesterol lipoprotein; LDL-C: low-density cholesterol lipoprotein | | | | |
| NSAIDS: non-steroidal anti-inflammatory drugs; | | | | |
